# Ankyrin-G Participates in I_Na_ Remodeling in Myocytes from the Border Zones of Infarcted Canine Heart

**DOI:** 10.1371/journal.pone.0078087

**Published:** 2013-10-14

**Authors:** Wen Dun, John S. Lowe, Patrick Wright, Thomas J. Hund, Peter J. Mohler, Penelope A. Boyden

**Affiliations:** 1 Department of Pharmacology, Center for Molecular Therapeutics, Columbia University, New York, New York, United States of America; 2 The Ohio State University Wexner Medical Center, The Dorothy M. Davis Heart & Lung Research Institute, The Ohio State University, Columbus, Ohio, United States of America; 3 Department of Biomedical Engineering,the Ohio State University College of Engineering, Columbus, Ohio, United States of America; 4 Department of Internal Medicine, the Ohio State University, Columbus, Ohio, United States of America; 5 Department of Biomedical Engineering, The Ohio State University College of Engineering, Columbus, Ohio, United States of America; Albert Einstein College of Medicine, United States of America

## Abstract

Cardiac Na channel remodeling provides a critical substrate for generation of reentrant arrhythmias in border zones of the infarcted canine heart. Recent studies show that Na_v_1.5 assembly and function are linked to ankyrin-G, gap, and mechanical junction proteins. In this study our objective is to expound the status of the cardiac Na channel, its interacting protein ankyrinG and the mechanical and gap junction proteins at two different times post infarction when arrhythmias are known to occur; that is, 48 hr and 5 day post coronary occlusion. Previous studies have shown the origins of arrhythmic events come from the subendocardial Purkinje and epicardial border zone. Our Purkinje cell (Pcell) voltage clamp study shows that I_Na_ and its kinetic parameters do not differ between Pcells from the subendocardium of the 48hr infarcted heart (IZPCs) and control non-infarcted Pcells (NZPCs). Immunostaining studies revealed that disturbances of Na_v_1.5 protein location with ankyrin-G are modest in 48 hr IZPCs. Therefore, Na current remodeling does not contribute to the abnormal conduction in the subendocardial border zone 48 hr post myocardial infarction as previously defined. In addition, immunohistochemical data show that Cx40/Cx43 co-localize at the intercalated disc (IDs) of control NZPCs but separate in IZPCs. At the same time, Purkinje cell desmoplakin and desmoglein2 immunostaining become diffuse while plakophilin2 and plakoglobin increase in abundance at IDs. In the epicardial border zone 5 days post myocardial infarction, immunoblot and immunocytochemical analyses showed that ankyrin-G protein expression is increased and re-localized to submembrane cell regions at a time when Na_v_1.5 function is decreased. Thus, Na_v_1.5 and ankyrin-G remodeling occur later after myocardial infarction compared to that of gap and mechanical junctional proteins. Gap and mechanical junctional proteins remodel in IZPCs early, perhaps to help maintain Na_v_1.5 subcellular location position and preserve its function soon after myocardial infarction.

## Introduction

 Impulse propagation in cardiac tissues depends on excitability of myocytes as well as electrical communication between myocytes. Excitability of a single cell is determined by Na_v_1.5, the major cardiac Na channel α subunit, its proper cell placement and function. Altered Na^+^ current (*I*_Na_) function in myocytes dispersed from the epicardial border zone (EBZ) of the 5 day infarcted canine heart plays an important role in the slowed conduction and subsequent reentry that occurs in this arrhythmic substrate [1-3]. Mohler et al reported that Na_v_1.5 proteins not only associate with ankyrin-G but also require ankyrin-G to target to the intercalated disc (ID) [4]. In fact, a human Brugada syndrome mutation in the ankyrin-G binding site of Na_v_1.5 causes aberrant sodium channel membrane trafficking [4]. We have previously shown that in the EBZ cells (IZs), there is marked structural remodeling of Na_v_1.5 [3]. What remains unknown are the changes in *I*_Na_, Na_v_1.5 and its adapter protein ankyrin-G, in the arrhythmic Purkinje cells that survive in the *subendocardial* Border zone (IZPCs) [5]. Therefore, we hypothesized that infarction induced remodeling of Na_v_1.5 is associated with changes in ankyrin-G in the canine epicardial and endocardial border zones. 

 More recently, Na_v_1.5 has been functionally and structurally linked with connexin43 (Cx43), plakophilin2 (PKP2), and ankyrin-G [6][7]. We therefore hypothesized that these proteins, as well as other desmosomal and disc proteins, may show early remodeling following myocardial infarction serving to further alter conduction. Thus here we use tissues and single cells dispersed from both the subendocardial and epicardial border zones post infarct to determine Na current function in relation to ankyrin-G. 

## Methods

This study was carried out in strict accordance with the recommendations in the Guide for the Care and Use of Laboratory Animals of the National Institutes of Health (Publication No. 85-23, 1996). The protocol for all animal procedures was approved by the Institutional Animal Care and Use Committee of Columbia University (Permit Number: AC-AAAD1067). Healthy mongrel male dogs (12 to 15 kg, 2 to 3 years old) were used in these studies. Under isoflurane anesthesia (30 mg/kg) and sterile conditions, myocardial infarction was produced by a 2-step total occlusion of the left coronary artery using the Harris procedure [8]. Dogs were treated with lidocaine (2 mg/kg IV) if multiple ventricular beats occurred at the time of the surgical procedure. Two or five days after myocardial infarction (MI) surgery, animals were sacrificed and cardiectomy performed. As before by visualization under microscope, thin slices of EBZ and tissues from a region remote to the EBZ (REMOTE) were obtained and used either for western blot assay and/or cell preparation [3] and immunocytochemistry. In addition, thin strands of subendocardial Purkinje fibers were dissected from the left ventricular (LV) subendocardium of the 48hr infarct and normal non-infarcted hearts and used to disperse cells (IZPCs and NZPCs) [9] for voltage clamp experiments and immunocytochemistry. Identification of Pcell phenotype was done as in previous studies [10]. Histologic evidence of the role of surviving Pcells and epicardial cells in border zones has been previously published [1]. Samples of the subendocardial Purkinje fiber tissues were embedded in O.C.T. (embedding medium, Tissue-Tek) and used for immunohistochemistry. 

### Preparation of single Purkinje cells

The enzymatic technique used to disaggregate Purkinje cells from control and infarcted hearts has been described [9]. In brief, small strips (4X2X2 mm) of left ventricular endocardium containing longitudinally oriented Purkinje fiber bundles were carefully dissected from larger preparations removed from specific regions in the control and infarcted hearts. The specific endocardial regions were identified carefully with a dissection microscope. After 8 to 10 of these mini-strips were prepared, the method of enzymatic disaggregation was used to make single Purkinje cells. After rinsing using Ca^2+^-free Krebs solution, these strips were transferred to the balanced salt solution (MEM, minimum essential medium concentrated amino acid and vitamins, GIBCO Grand Island New York, pH 6.72) with collagenase type II (Worthington) 0.2% 310 units plus 0.3% 315 units and incubated in a shaking bath (38°C) for about 40 min. At this stage the connective tissue sheath around the Purkinje myocytes should be fuzzy and sticky. Enzyme –containing solution was carefully removed and then contents were washed twice with a high K^+^ saline solution (Purkinje disaggregation solution, pH 6.72). The contents were placed in the shaker bath (38°C) and allowed to recover for 10 minutes. Individual cells were dispersed in Purkinje disaggregation solution by gentle hand pipetting. After the cells were harvested, the tube containing the supernatant was centrifuged for 40-60 sec. This produced a loose pellet. The final supernatant was extracted and discarded and this loose pellet was resuspended in MEM solution (pH=7.3). Ca^2+^ concentration of the cell resuspension solution was gradually increased and the cells were finally kept in 0.5 mM Ca^2+^ MEM solution.

### Preparation of single EBZ and Remote cells from epicardial layer

Single calcium-tolerant cells were dispersed from EBZ (IZs) and Remote sections of 5-day infarcted canine hearts using a modification of our previously described method [1]. Briefly, the tissue was rinsed twice in a Ca^2+^-free solution containing (in mM) NaCl 115, KCl 5, sucrose 35, dextrose 10, *N*-2-hydroxyethylpiperazine-*N*’-2-ethanosulfonic acid (HEPES) 10, taurine 4, pH 6.95, to remove blood. Then, it was triturated in 20 ml of enzyme-containing solution (collagenase type II from Worthington Biochemical, Lakewood, NJ; 0.38 mg/ml, 36-37°C) for 30 min twice, after each which, the solution was decanted and discarded. Note that only in the first time, the solution contained 0.01 mg/ml protease (Sigma). The following six to seven time triturations were each done for 15 min. Each time, the solution was centrifuged at 500 rpm for 3 min to collect the supernatant and dispersed cells. Resuspension solution was changed every 30min for solution containing increasing concentration of Ca^2+^ (50 to 500 µmol/L). With this procedure, the viable cell yield was approximately 30-40%. 

### Immunoblot

Canine cardiac tissue was flash frozen with liquid N_2_ and ground into a fine powder using a chilled mortar and pestle as described [11] [12]. The resulting powder was then resuspended in homogenization buffer (1 mM NaHCO_3_, 5mM EDTA, 1 mM EGTA, 2 mM Na_3_VO_4_, 1 mM NaF, 1 mM PMSF, and protease inhibitor cocktail [Sigma]) and further homogenized using a chilled Dounce homogenizer. Samples were flash frozen in liquid N_2_ and stored at -80°C for immunoblots. Following quantification, EBZ and Remote lysates were analyzed on Mini-PROTEAN tetra cell (BioRad) on a 4-15% precast TGX gel (BioRad) in Tris/Glycine/SDS Buffer (BioRad). For each lysate, 15 µl of sample (volumes were normalized for varying protein concentrations) was loaded with 15 µl of a 20:1 mixture of Laemmli sample buffer (BioRad) and β-mercaptoethanol (BME) as described [13]. Samples were boiled at 100°C for 5 min prior to loading. Gels were transferred to a nitrocellulose membrane using the Mini-PROTEAN tetra cell (BioRad) in Tris/Glycine buffer with 10% methanol (v/v, BioRad). Membranes were blocked for 1 hour at room temperature using a 3% BSA solution and incubated with primary antibody overnight at 4°C. Antibodies included: ankyrin-G ([14] [15]); Na_v_1.5 ([14][15][16]). Densitometry was performed using Adobe Photoshop software and all data was normalized to GAPDH levels present in each sample. Band densities from immunoblots were measured with Adobe Photoshop 8.0, normalized to actin or GAPDH.

### Real-time PCR

#### mRNA levels

mRNA was evaluated as previously described [11] [12]. Total RNA from remote and EBZ was isolated using the RNeasy mini kit (Qiagen). Isolated RNA concentrations were calculated using spectroscopy (260 nm). 100-500 ng of total RNA was used for reverse transcription using SuperScript III (Invitrogen). PCR was performed on 4 µL of each cDNA reaction using TAQ polymerase (New England Biolabs) per the directions of the manufacturer.

### Immunostaining and confocal microscopy

For immunocytochemistry, single cells dispersed from both EBZ (IZs) and Remote and LV subendocardium (IZPCs and NZPCs) were plated on laminin-coated glass chamber slides. For immunohistochemistry, 5 µm tissue cryosections from 48 hour infarct Purkinje fibers (IZPFs) and non-infarct Purkinje fibers (NZPFs) were collected on coverslips. Both single cells and tissue sections were fixed with 4% paraformaldehyde for 15 minutes, rinsed with PBS (Sigma), permeabilized by 0.7% Triton X-100 (Sigma) for 20 minutes, blocked in the serum for 30 minutes, and then incubated with primary antibodies overnight at 4°C. The single cells or tissue sections were rinsed in PBS, incubated with 1:400 Alexa Fluor 488-conjugated or/and 594-conjugated IgG (Molecular Probes) for 1.5 hours at room temperature, and rinsed in PBS. Coverslips were mounted on slides by using aqueous mounting medium (Biomeda Corp, Foster City, CA). Signals were viewed by using Zeiss LSM 510 META laser-scanning confocal system (488 nM and 594 nM excitation). Care was taken to view images from noninfarcted and infarct preparations on same scope on same day. 

### Antibodies

Ankyrin-G (diluted 1:200) and Na_v_1.5 (1:500) antibodies have been described [4,15,16].Additional antibodies include rabbit anti-Connexin 40 (1:200, Alpha Diagnostic), mouse anti-Connexin 43 (1:200, Millipore), rabbit anti-N-Cadherin (1:100, abcam), mouse anti-Plakophilin2 (Progen), mouse anti-Desmoplakin 1&2 (Progen), rabbit anti-Desmoglein 2 (1:100, Progen), and goat anti-Plakoglobin (1:100, Sigma). 

### Electrophysiology

Single cells were placed at the bottom of a 0.5-ml tissue chamber, which had been mounted on the stage of a Nikon inverted microscope (Nikon Diaphot, Tokyo, Japan). The myocytes were superfused (2–3 ml/min) with Tyrode’s solution containing (in mmol/L) NaCl 137, NaHCO_3_ 24, NaH_2_PO_4_ 1.8, MgCl_2_ 0.5, CaCl_2_ 2.0, KCl 4.0, and dextrose 5.5 (pH7.4). Patch pipettes were made from borosilicate thin-wall glass and pipette resistances ranged between 1.0 and 1.5 M when filled with an internal solution containing (in mmol/l) CsOH 125, aspartic acid 125, tetraethylammonium chloride 20, HEPES 10, Mg-ATP 5, EGTA 10, and phosphocreatine 3.6 (pH 7.3 with CsOH). After the formation of the gigaohm seal, the cell membrane under the pipette tip was ruptured by a brief increase in suction, forming the whole cell recording configuration. A period of 5-10 min was then allowed for intracellular dialysis to begin before switching to the external recording solution containing (in mmol/L) NaCl 3 for Purkinje cells, MgCl_2_ 1.2, CaCl_2_ 1.8, tetraethylammonium chloride 80, CsCl 5, HEPES 20, glucose 11, 4-aminopyridine 3.0, and MnCl_2_ 2.0 (pH 7.3 with CsOH). Data acquisition from all cells studied was done at the same time after whole cell. In this way cells from normal and diseased tissues could be compared. Temperature was monitored continuously and maintained at 19.0 ± 0.5°C for proper voltage control. With this combination of external and internal solutions, *I*_Na_ would be of manageable size and isolated from other possible contaminating currents. Whole cell *I*_Na_ was obtained by subtracting the traces elicited with comparable voltage steps containing no current (using prepulse to inactivate the Na^+^ channels) from the raw current traces. In this way, the cell capacitance and linear leakage, if present, were subtracted. Peak current density in cells from the different groups, *I-V* data, activation data, steady-state availability (I/I_max_), the time course of inactivation directly from the closed state that is described by two time constants (_1_ and _2_), and time course of recovery of *I*_Na_ from steady-state inactivation were assessed by using previously published protocols [17]. All data were collected at similar times after whole-cell membrane rupture.

### Statistics

Values represent mean ± SD (or mean ± SE) A value of *P* < 0.05 was considered statistically significant. For a two-sample comparison, an unpaired *t*-test was used to compare a single mean value between the two independent cell groups. For multiple comparisons, an ANOVA was used to determine if the sample mean values between groups were significantly different from each other. If so, a modified *t*-test with Bonferroni correction was used. 

## Results

### Subendocardial Purkinje cells; Na^+^ current form and function

Following 24-48 hrs (subacute phase of infarction) post occlusion, delayed spontaneous arrhythmias of ventricular origin (subendocardial Purkinjes) occur in our experimental model as well as may have counterparts in humans [18]. Generally, by 48 hrs post occlusion action potentials of the subendocardial Purkinje cells (Pcells) show reduced resting potentials as well as an increase in total time of repolarization. Importantly, the abnormalities in the resting and action potentials (APs) of subendocardial Purkinje fibers surviving in the 24-48 hr infarcted heart persist even after they are enzymatically disaggregated and studied as single myocytes called IZPCs [10]. 

**Figure 1:**
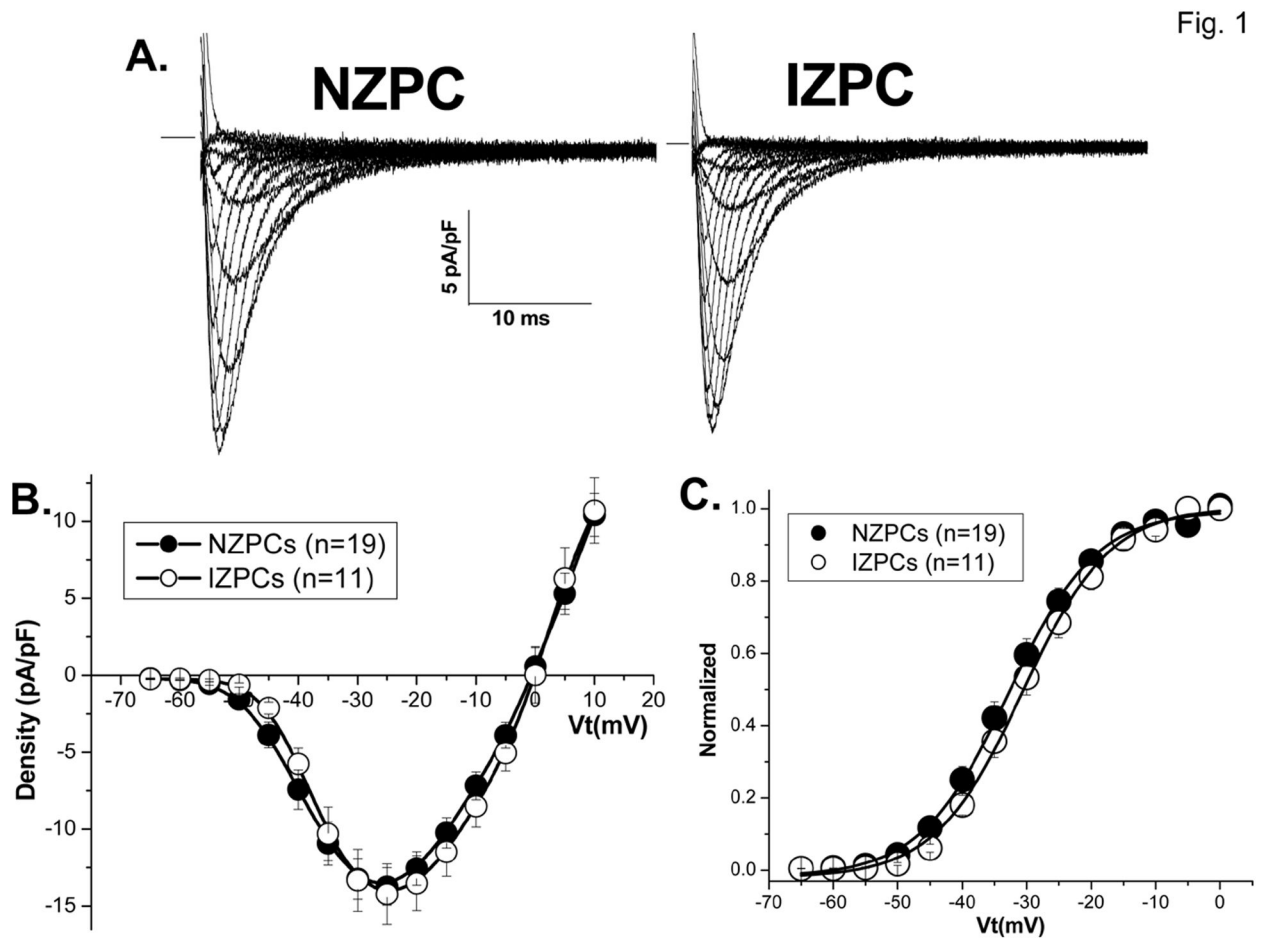
Sodium currents do not differ between NZPCs and IZPCS. Family of tracings of *I*_Na_ in NZPC (Panel A, Left) and IZPC (Panel A, Right). *I*_Na_s were elicited from a *V*_H_ of -100 mV to various levels of *V*t (-65 to +10 mV). The calibration bar is inserted. Panel B: *I*_Na_ density-voltage relationships in NZPCs (n=19) and IZPCs (n=11). There is no significant difference in the peak currents at voltage -25 mV between NZPCs and IZPCs (*P*>0.05). Panel C: Steady-state activation of *I*_Na_ in NZPCs and IZPCs. Curves drawn represent Boltzmann equation using average of fit values (see Table 1). All data were collected at 23-25 min after whole-cell membrane rupture.

**Figure 2:**
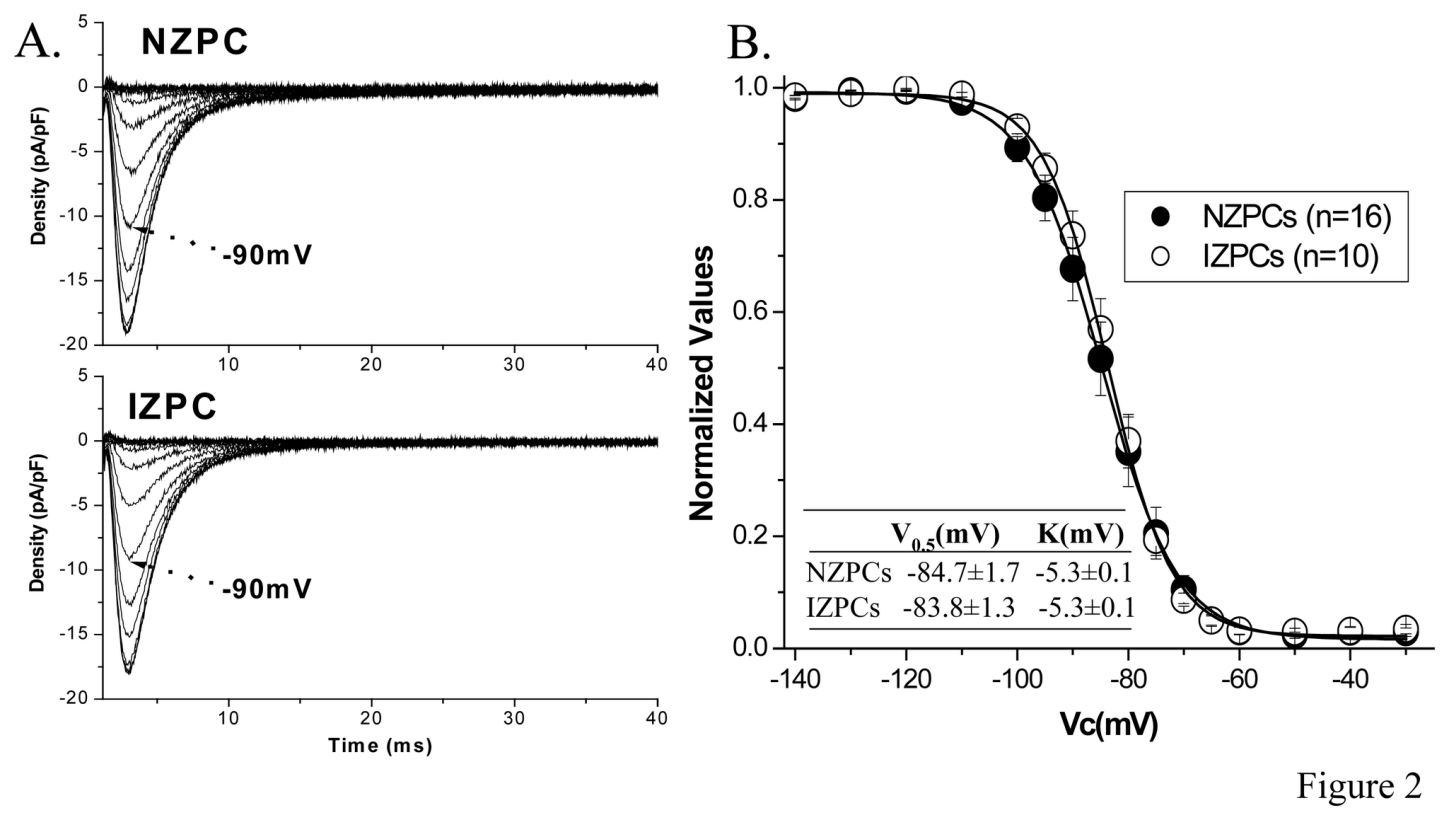
Sodium current inactivation does differ between the two groups. Steady state inactivation of *I*_Na_ in NZPCs and IZPCs. Panel A, the original tracings of *I*_Na_ obtained during the “steady state” inactivation protocol in NZPC (top) and IZPC (bottom) after subtraction of membrane capacity and linear leakage. Arrow indicates *I*_Na_ after conditioning potential (V_c_) to -90 mV. Panel B shows average I/I_max_ curves constructed using data recorded with the same protocol in both cell types. The current amplitude elicited by the test pulse at each prepulse voltage was normalized to the maximal current obtained after prepulse voltage to -140 mV. The I/I_max_ curves were created by plotting the normalized current against V_c_. V_0.5_ was -84.4±1.7 mV in NZPCs (n=16) and -83.8±1.3 in IZPCs (n=10) (P>0.05).

**Figure 3:**
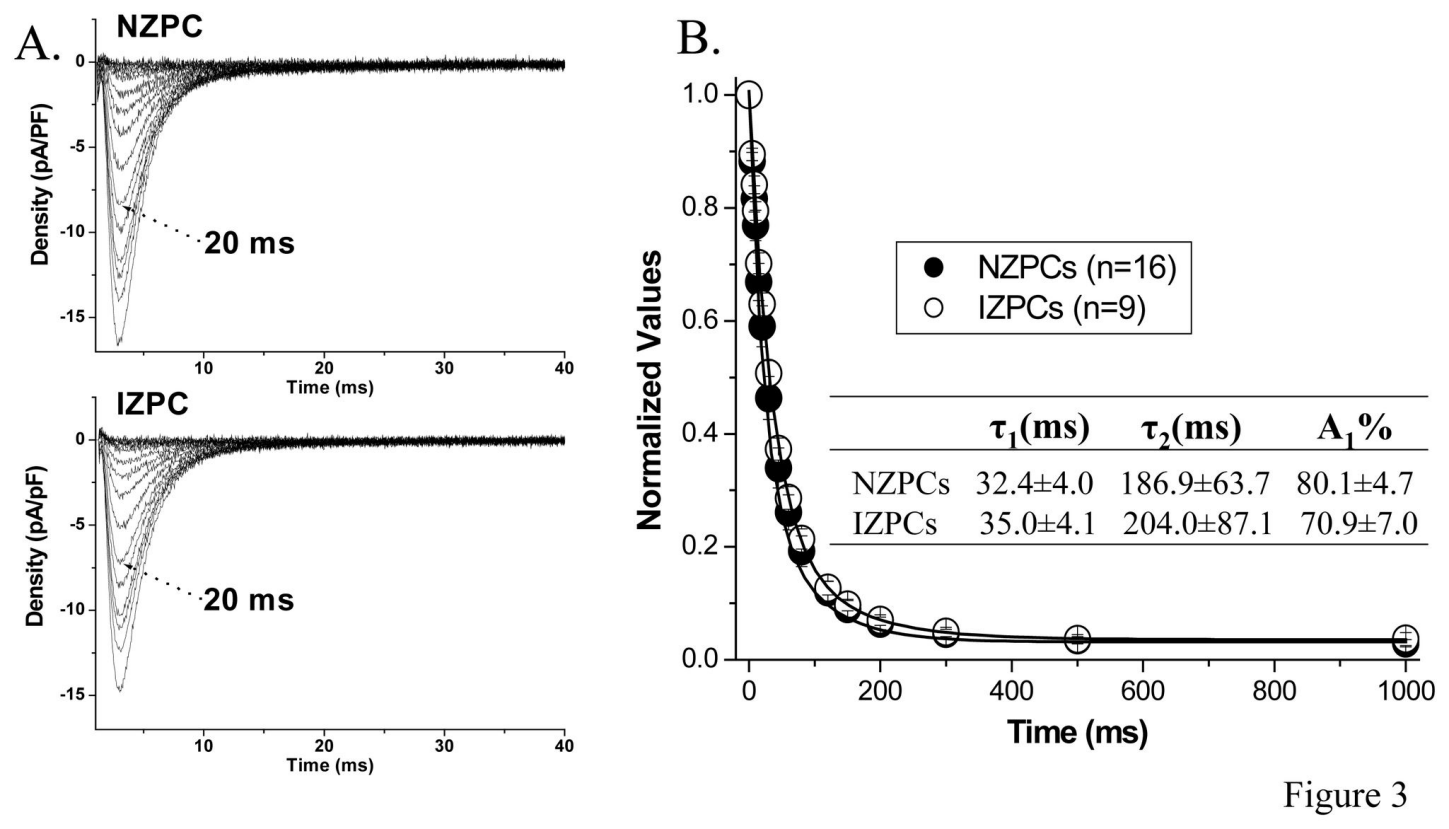
Rate of development of inactivation of *I*_Na_ from depolarized conditioning potential (-60 mV) does differ between the two groups . Panel A shows *I*_Na_ tracings from NZPC (top) and IZPC (bottom) obtained during protocol. The test *I*_Na_ (V_H_=-100 to -25 mV) obtained after prepulses to -60 mV of variable duration are superimposed. Note that there were no significant differences between NZPC and IZPC. Panel B, *I*_Na_ amplitude of the test pulse is normalized to maximal *I*_Na_ (*I*_max_, obtained with 0-ms conditioning pulse). The time course of *I*_Na_ inactivation was best described by a bi-exponential function (see inset).

**Figure 4:**
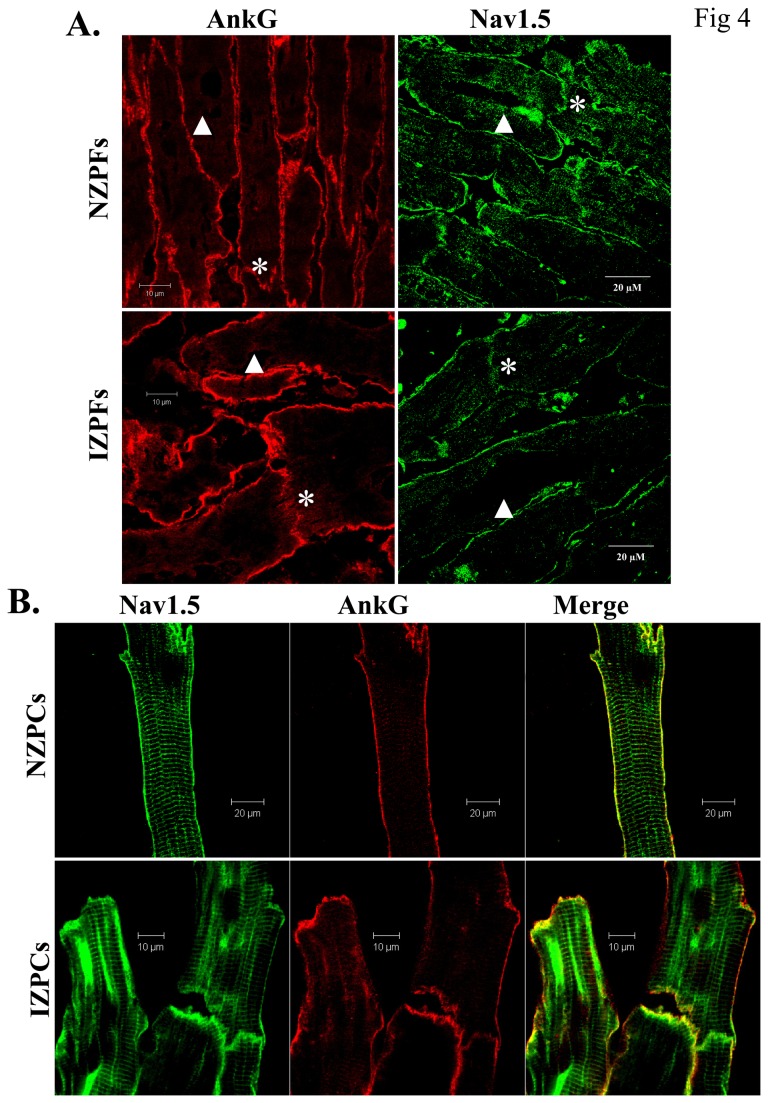
Co-staining of Nav1.5 and ankyrin-G in non-infarcted and infarcted Purkinje cells. Panel **A**. Distribution of ankyrin-G and Na_v_1.5 protein in NZPFs (upper) and IZPFs (lower). Fixed Purkinje fibers were cryostat-sectioned (5 µm) longitudinally and labeled with anti-ankyrin-G and Na_v_1.5 antibodies. Note that Na_v_1.5 is on cell surface and IDs in both NZPFs and IZPFs. Very few side-to-side junctions are observed in Purkinjes (Arrowheads) Panel **B**, Co-staining of Na_v_1.5 with ankyrin-G (AnkG) in NZPC (**upper**) and IZPCs (lower). Co-localization of ankyrin-G and Na_v_1.5 is similar in NZPCs and IZPCs.

By virtue of the loss of resting potential, there would be a predictable change in V_max_ of the subendocardial PCells that survive post MI. In fact, IZPCs have markedly reduced AP amplitudes and V_max_ [19]. Here we report that average peak *I*_Na_ density of NZPCs did not differ from that of IZPCs and activation curves were similar in two cell types (Figure **1** and Table **1**). *I*_Na_ recovery from inactivation in NZPCs and IZPCs do not differ (Table **2**). Steady-state inactivation curves (Figure **2**) and the time course of closed-state inactivation (Figure **3**) also did not differ between NZPCs and IZPCs. Thus conduction abnormalities in IZPCs of subendocardial border zones are not due to abnormal Na current function. Furthermore, disturbances of Na_v_1.5 protein location with its adapter partner ankyrin-G are modest in 48 hr IZPCs (Figure **4A, B**)**.**

**Table 1:** Steady-state activation of *I*_Na_ in NZPCs and IZPCs.

	Peak density(pA/pF)	V_0.5_(mV)	k(mV)	E_rev_(mV)
NZPCs(n=19)	14.6±1.3	-32.4±1.3	6.2±0.2	0.55±1.3
IZPCs(n=11)	14.7±1.9	-30.4±1.3	6.4±0.4	0.27±1.5

NZPCs indicate Purkinje myocytes dispersed from subendocardium the left ventricle of non-infarcted hearts. IZPCs indicate Purkinje myocytes from subendocardium of 48-hour infarcted hearts. Values for the half-maximum voltage, V_0.5_, and the slope factor, k, were obtained by best-fitting the data from each cell to a Boltzmann function. E_rev_ indicates reversal potential of I-V curves of *I*_Na_. All data are presented as mean ± SE.

**Table 2:** Time constant of recovery of *I*_Na_ from inactivation in NZPCs and IZPCs.

	**Recovery at V_h_-100mV**		**Recovery at V_h_-90mV**	
	NZPCs(18)	IZPCs(11)	NZPCs(16)	IZPCs(10)
τ_1_ (ms)	26.9±2.5	28.9±3.9	53.1±5.0	55.8±8.1
τ_2_ (ms)	402.5±21.3	443.1±21.4	521.8±50.2	600.2±85.0
A_1_%	60.4±2.5	57.4±4.3	63.2±2.5	55.6±3.1

NZPCs indicate Purkinje myocytes dispersed from subendocardium the left ventricle of noninfarcted hearts. IZPCs indicate Purkinje myocytes from subendocardium of 48-hour infarcted hearts. Time course of recovery was best described by a biexponential function. τ_1_ was fast time constant of recovery from inactivation and τ_2_ was slow time constant of recovery from inactivation, A_1_% indicated amplitude of the fast-component time constant normalized to total amplitude. All data are presented as mean ± SE. V_H_ indicates holding voltage.

### Gap and Mechanical junctions in Subendocardial Border Purkinje

**Figure 5:**
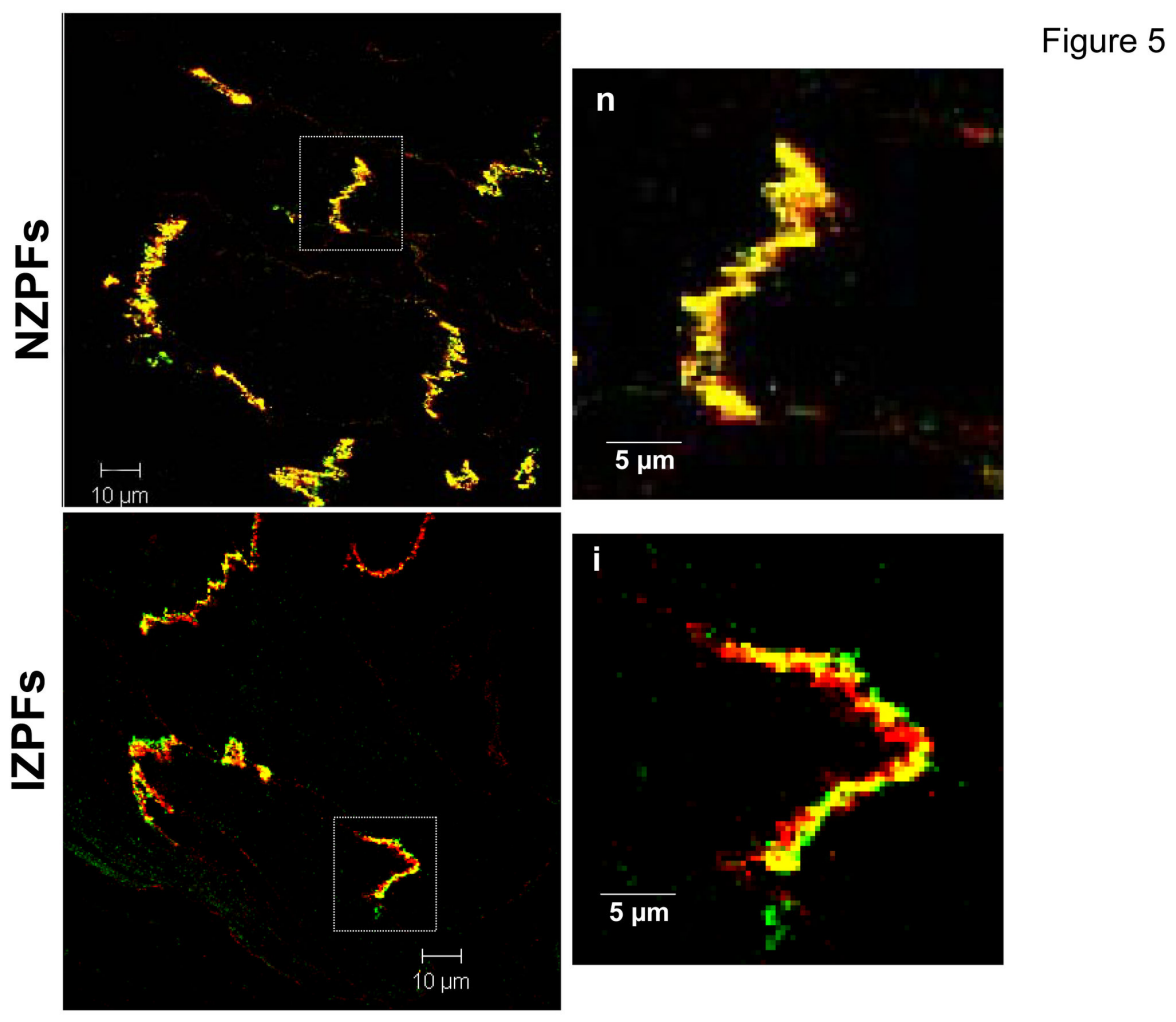
Co-staining of Cx40 (Green) and Cx43 (Red) in NZPFS (upper) and IZPFs (lower). The enlarged images from the white rectangles of NZPFs and IZPFs are shown to the right, respectively. In NZPFs, Cx40 and Cx43 are uniformly and tightly colocalized at IDs, showing concentrated yellow merge sites. However, in IZPFs, individual bands of Cx40 and Cx43 are separated, exhibiting discrete red (Cx43), green (Cx40) bands. Yellow (merged) bands at IDs still exist.

**Figure 6:**
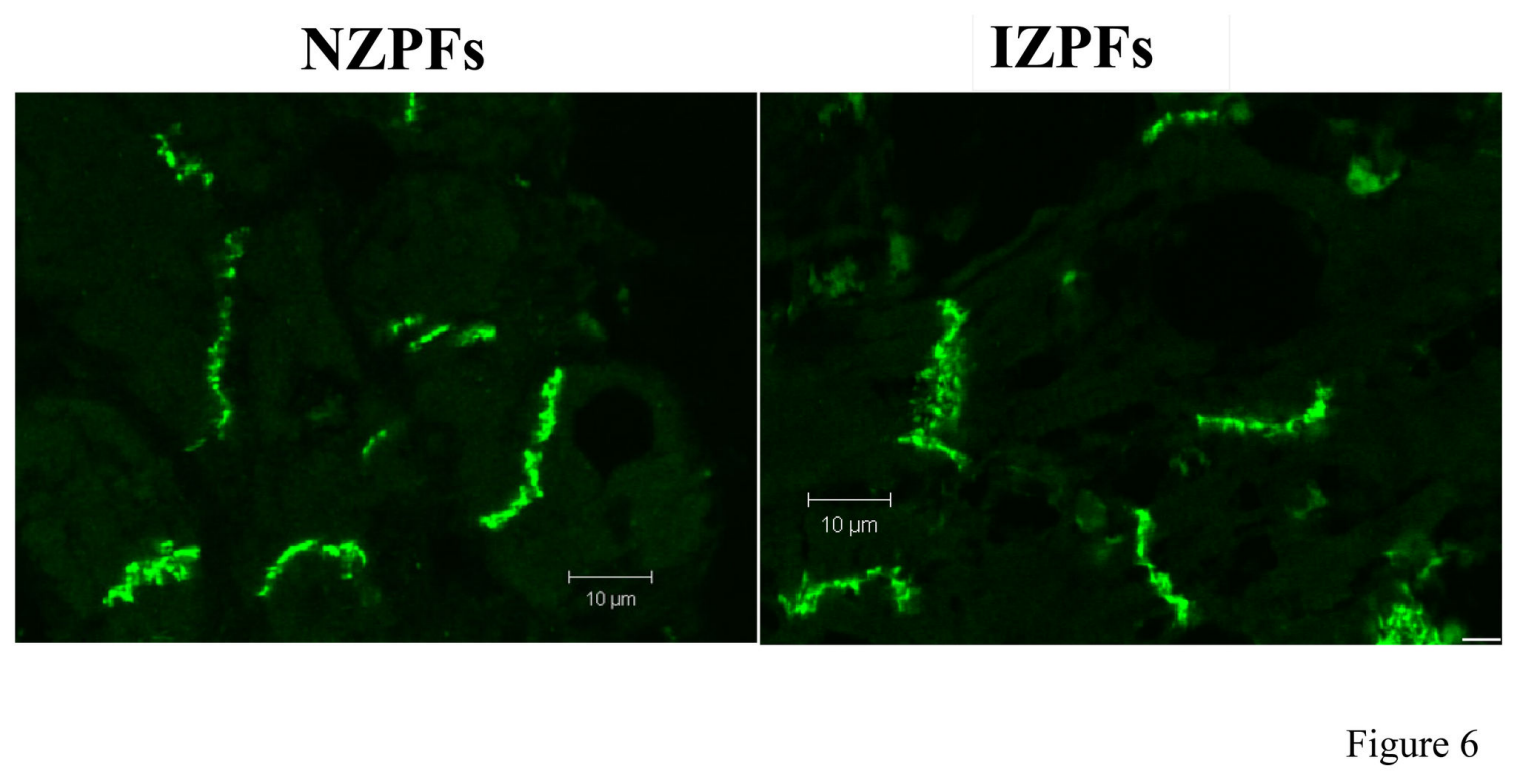
N-Cadherin expression and localization in NZPFs and IZPFs. N-cadherin signals in both NZPFs and IZPFs were located at the IDs.

 Gap junctional proteins are obvious logical candidates to contribute to abnormal electrical properties in Purkinjes of the subendocardial border zone. The major connexin protein in Purkinje cells is connexin 40 (Cx40). Interestingly, unlike the area *Composita* of ovine and bovine Purkinje cells [20][21], Cx40 cell localization is quite polarized in canine Purkinjes, existing at the cell ends in both normal (NZPFs) and infarct (IZPFs) tissues (Figure **5**) and single cells (not shown). In NZPFs, Cx40 and Cx43 co-localize at the IDs appearing as yellow bands (Figure **5**). On rare occasions, a side-to-side junction is observed. In IZPFs, there is little Cx40 lateralization, yet while co-localization of Cx40/Cx43 is still evident, clear bands of solo Cx40 and Cx43 are present (Figure **5** **lower**). Thus the heteromeric assembly of connexin proteins in NZPFs is remodeled in IZPFs to bands of homomeric gap junctional channels. Note very little connexin redistribution is evident and N-cadherin localization in both cell types is unchanged (Figure **6**).

**Figure 7:**
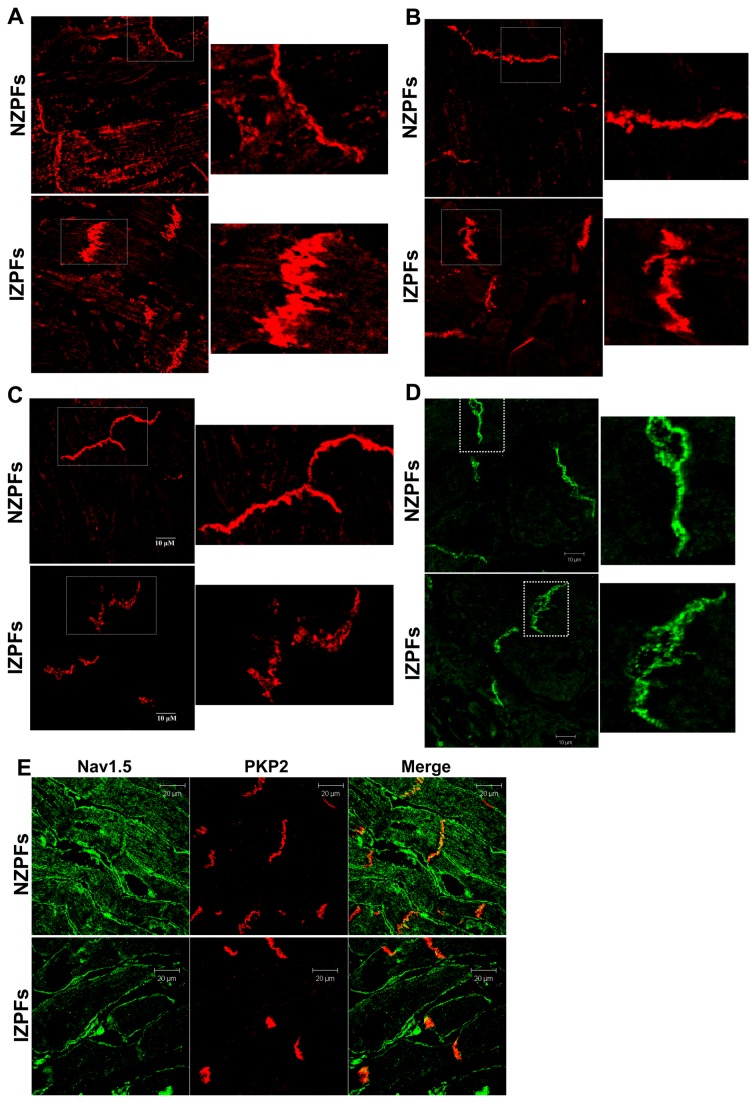
Expression and localization of plakophilin2 (PKP2, panel A), plakoglobin (PKG, panel B), desmoplakin (DP, panel C) and desmoglein-2 (Dsg2, panel D) in NZPFs (upper) and IZPFs (lower). The enlarged images of white rectangles in NZPFs and IZPFs are shown to the right. Immunofluorescence signals show that PKP2 (**A**) and PKG (**B**) thickens at some sites of the IDs in IZPFs, while there is a lower abundance and diffusion of DP(C) and Dsg2 (D) at the IDs in IZPFs. Panel **E**, Co-staining for Na_v_1.5 (Green) with PKP2 (Red) in NZPFs (Upper) and IZPFs (lower). PKP2 and Na_v_1.5 colocalized at IDs in both NZPFs and IZPFs.

 As expected, Pcells are enriched in desmosomal proteins. In frozen sections of NZPFs and IZPFs, proteins of the mechanical junctions, desmoplakin (DP), desmoglein2 (Dsg2), Plakophilin2 (PKP2) and plakoglobin (PKG) were remodeled in IZPFs (Figure **7A-D**). In IZPFs, DP and Dsg2 become diffuse at ID while PKP2 and PKG increase in abundance at ID, particularly PKP2. Interestingly, in co-staining of PKP2 with Na_v_1.5 in IZPFs, we observed that Na_v_1.5 is at cell surface and IDs where there is pronounced abundance of PKP2 (Figure **7E**). Together, our data demonstrate that in remodeled adult Purkinje cells surviving in the infarcted heart, Na channel protein/function is not yet altered and expression of the adapter protein ankyrin-G is still robust. Gap junctional proteins are altered in assembly but have not redistributed. Finally, mechanical junction proteins display notable plasticity, perhaps to “stabilize” the cells following ischemic damage. 

### Na_v_1.5 protein of cells of EBZ decreases as ankyrin-G increases

**Figure 8:**
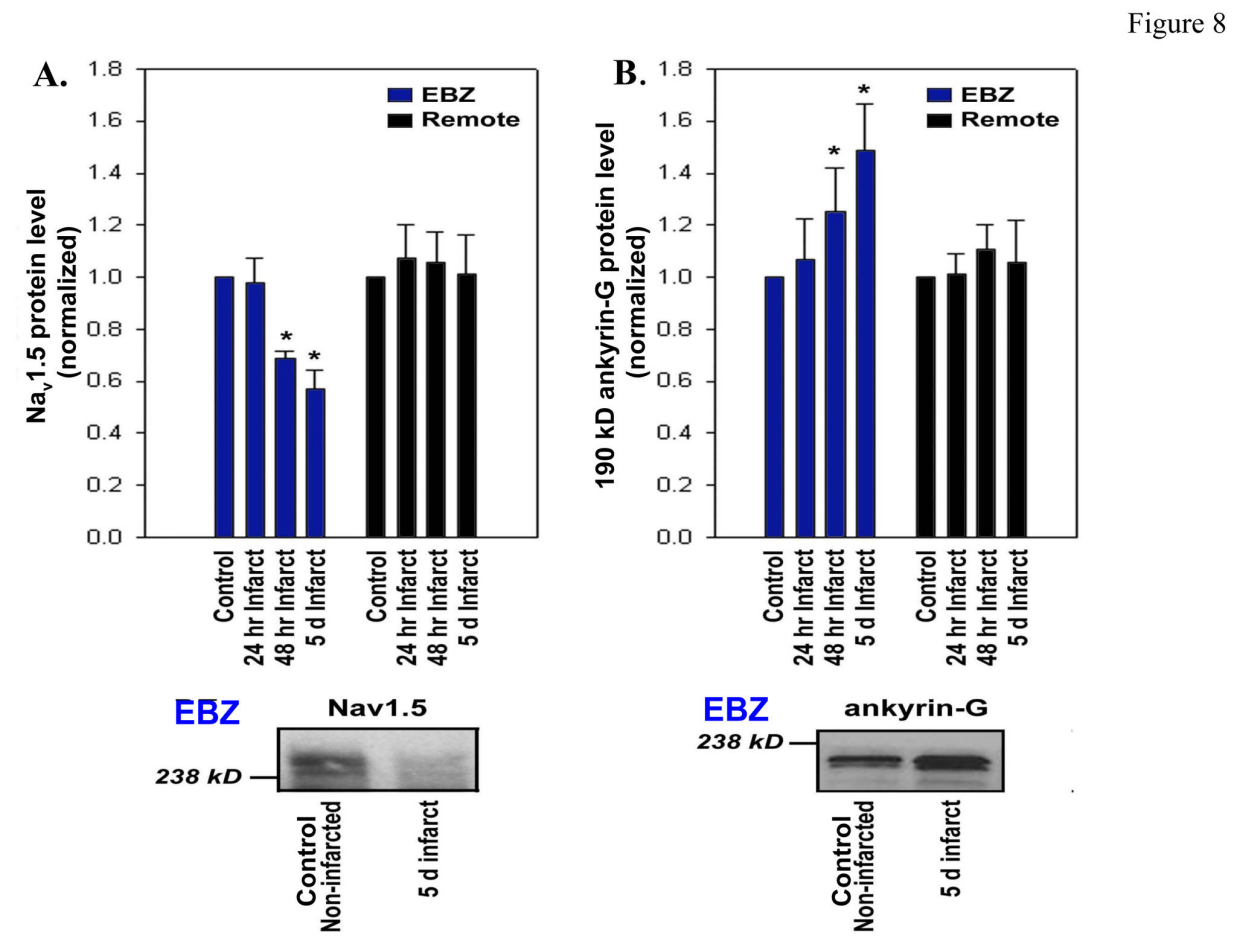
Detection of Na_v_1.5 and ankyrin-G by immunoblot. Relative expression of Na_v_1.5 (Panel **A**) and 190 kD ankyrin-G (Panel **B**) in control (non-infarcted hearts) and post infarct canine epicardial tissue.  Tissues were collected from infarcted hearts (both EBZ and Remote) at post occlusion 24 hr, 48 hr, and 5 days.  Cell lysates were prepared and equal quantities of protein were analyzed by SDS-PAGE and blotted using affinity-purified Ig prepared against 190 kD ankyrin-G, Na_v_1.5, or a loading control. Linear band intensities were normalized, averaged and plotted as levels relative to control samples (N=4).  Means ± SEM. An example of blots is shown below each graph, respectively.

Figure **8A** shows that there is a decrease in Na_v_1.5 protein in EBZ (blue bars) following coronary artery occlusion. By 48 hrs post-occlusion, this decrease is significantly different from control LV epicardium from noninfarcted heart tissues. By 5 days post occlusion, there is 42% decrease in Na_v_1.5 protein. We have previously shown that at this time post occlusion, *I*_Na_ is reduced in density by 44% [3]. Notably there is no change in Na_v_1.5 in LV tissues remote from the infarct (Figure **8A**, black bars). An analysis of the same proteins for 190kD ankyrin-G reveals that there is an *increase* in levels post coronary artery occlusion (Figure **8B**, blue bars). The greatest increase occurs at 5 days post MI (~50% increase) when Na_v_1.5 is markedly decreased (Figure **8A**) and function impaired [3]. Surprisingly, there is no change in ankyrin-G in tissue samples remote from the infarct (Figure **8B**, black bars). Finally, we observed no significant difference in ankyrin-G mRNA levels between normal, 48 hour, 2 day or 5 day border zone or remote samples (p=N.S.; data not shown). 

### Structural remodeling of Nav1.5 and ankyrin-G in cells from 5 day EBZ

**Figure 9:**
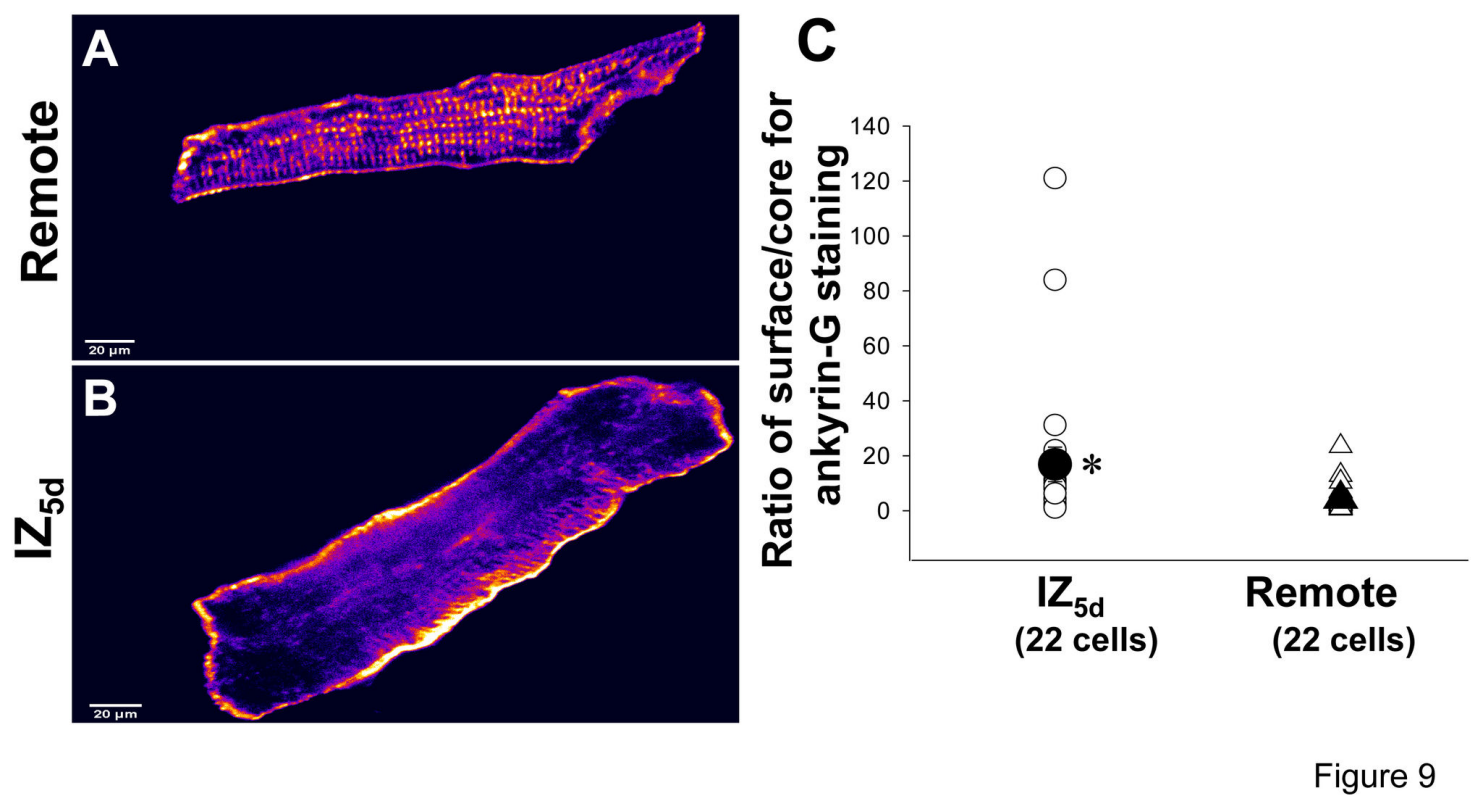
Immunolocalization of ankyrin-G in 5 day IZ (panel A) and Remote cells (panel B). The altered ankyrin-G staining appears to increase in signal just below membrane in IZ. Panel **C**: Ratio of surface/core of ankyrin-G staining measured in IZ and Remote. Open symbols: individual measurements. See text for details Filled symbols and vertical bars: means and SE. *P<0.05 vs Remote.

 In Baba et al [3] we systematically analyzed the form (cellular distribution) and function of the Na channel protein Na_v_1.5 in single cells dispersed from the 5 day EBZ. These immunocytochemistry experiments indicated that the α-subunit, Na_v_1.5 protein, is reduced at the IZ cell surface but remains at the ID region which is consistent with measured reduced whole cell peak *I*_Na_. We therefore tested whether this loss of Na_v_1.5 was due to an absence of ankyrin-G near the IZ cell surface using single cells from both the Remote and 5 day EBZ regions. In remote cells, ankyrin-G staining was localized to IDs, but also secondarily found at t-tubular and lateral membranes (Figure **9A**) similar to what has been described for normal mice myocytes by our group and others [11]. In contrast, ankyrin-G immunostaining in IZs was variable with many cells showing increased immunosignal near cell membrane (Figure **9B**). In fact, the ratio of surface/core ankyrin-G staining in 5 day IZs (16.9±6.3, n=22) was significantly greater than that in remote cells (4.3±1.1, n=22; P<0.05) (Figure **9C**). 

 Thus, with time post occlusion, an increase in amount of ankyrin-G appears to accumulate near or at the surface of surviving IZs. In sum, ankyrin-G protein expression increases and translocates to a submembrane region in cells that survive in the infarcted heart at a time when Na_v_1.5 protein decreases and *I*_Na_ function is depressed. Thus, in the acquired remodeling of *I*_Na_ in EBZ 5 day cells, ankyrin-G is clearly associated with myocyte electrical remodeling. 

## Discussion

Dysfunction in Na_v_1.5, ankyrin-G, Cx43, Cx40 and desmosomal proteins are linked to potentially fatal human congenital arrhythmias. Much less is known about status of these proteins in acquired human diseases. Here we have used a well described canine model of arrhythmias that occur post MI. Ion channel remodeling in different cell types (canine Purkinje and epicardial cells) occurs continually during the time post coronary artery occlusion [18]. In this study we focused on the status of the cardiac Na channel, its interacting protein ankyrin-G and the mechanical and gap junctional proteins at two different time points. 

48hrs after the MI, arrhythmias originate in the surviving subendocardial Purkinje cells which from our data shown above, have no alteration as yet of the Na channel protein (and function) and ankyrin-G. However, by this time point, gap junctional proteins Cx40/Cx43 have begun to change but not yet lateralized as has been described for the 5 day EBZ cells [22]. Thus slowed conduction of this border zone region [1] is more likely due to altered Purkinje I_K1_ [23]. Interestingly, we define here that in canine Purkinje cells from both normal and diseased hearts, adherens junctions are polar, unlike their ovine/bovine counterparts [20][21]. Furthermore some junctional proteins begin to increase in abundance but remain at Purkinje IDs. In particular, we show PKP2 has increased abundance in IZPFs but remains colocalized with Na_v_1.5 at IDs (Figure **7E**). Thus gap and mechanical junctional protein remodeling in infarct Purkinje cells occurs in the absence of a loss of Na channel structure and function by 48 hrs post occlusion. This was unexpected since recent findings in mice have suggested that the function of the Na_v_ channel at IDs depends on the desmosomal protein Plakophilin2 (PKP2). If PKP2 is silenced Na current function diminishes [7]. Therefore arrhythmias occurring in the Purkinje subendocardial border zone may not be directly related to Na channel protein dysfunction.

At 5 days after MI, a sharp epicardial border has formed in this canine model [18]. We and others have defined several ion channels that are remodeled in myocytes of the EBZ. In fact we have shown that cellular Na current function is markedly changed and with computer simulations, these changes in Na currents underlie post repolarization refractoriness and help to form lines of functional block. With a correctly timed premature stimulus, sustained reentry occurs around the lines of functional block [3].

 A finding from these new data is that in contrast to what we had hypothesized, ankyrin-G protein increases and resides under the cell membrane and IDs in the cells that survive in the 5 day epicardial border zone (IZs). In fact ankyrin-G protein expression increases at a time of loss of Na channel function and structural reorganization [3]. While there is an obvious reserve of Na channels in the heart, optimal function will depend on their location and anchoring by ankyrin-G. In the acute phase of remodeling of ischemia, it is likely ankyrin-G levels are acutely reduced by the calcium-activated, calcium-dependent calpain, similar to ankyrin-B [24]. Thus, we suggest that following 48 hours, ankyrin-G levels recover and stabilize in an effort to actively regulate Na_v_1.5 channel protein recruitment to sarcolemmal membrane. 
